# Supplementary material for: NIH/3T3 Fibroblasts Selectively Activate T Cells Specific for Posttranslationally Modified Collagen Type II
Source: Int J Mol Sci. 2023 Jun 28;24(13):10811. doi: 10.3390/ijms241310811 (PMC10341784; doi:10.3390/ijms241310811)
Supplement: Supplementary file 1 [file ijms-24-10811-s001.zip › ijms-2464595-supplementary.pdf]

International Journal of
Molecular Sciences

MDPI

Supplementary Materials

NIH/3T3 Fibroblasts Selectively Activate T Cells Specific for Posttranslationally Modified Collagen Type II

Balik Dzhambazov [1,*], Tsvetelina Batsalova [1], Patrick Merky [2], Franziska Lange [3] and Rikard Holmdahl [4]

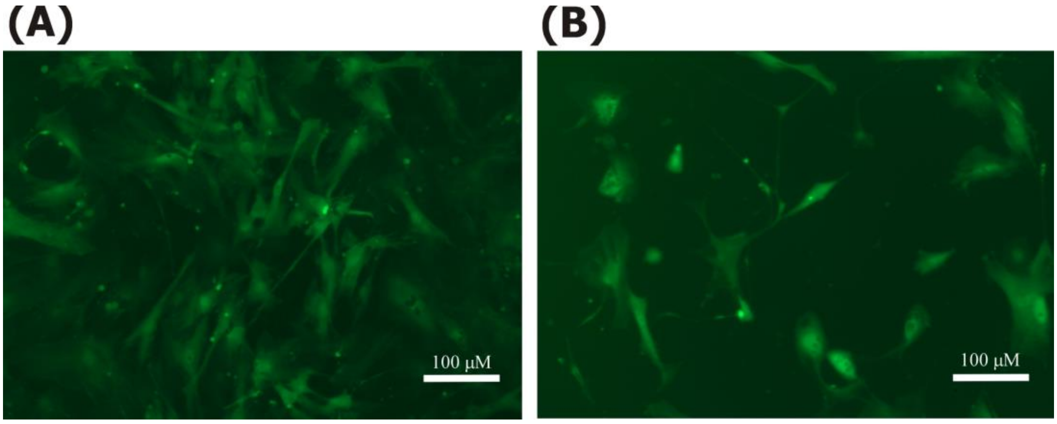

Figure S1. Expression of the Aq molecule on QB dermal fibroblasts **(A)** and QB synovial fibroblasts **(B).** After adhesion for 24 hours, the cells were stained with biotin labeled anti-mouse Aq (clone KH116) and FITC Streptavidin for 15 minutes at 37°C in dark. Photomicrographs show fluorescence from the respective area. Bar 100 µm.

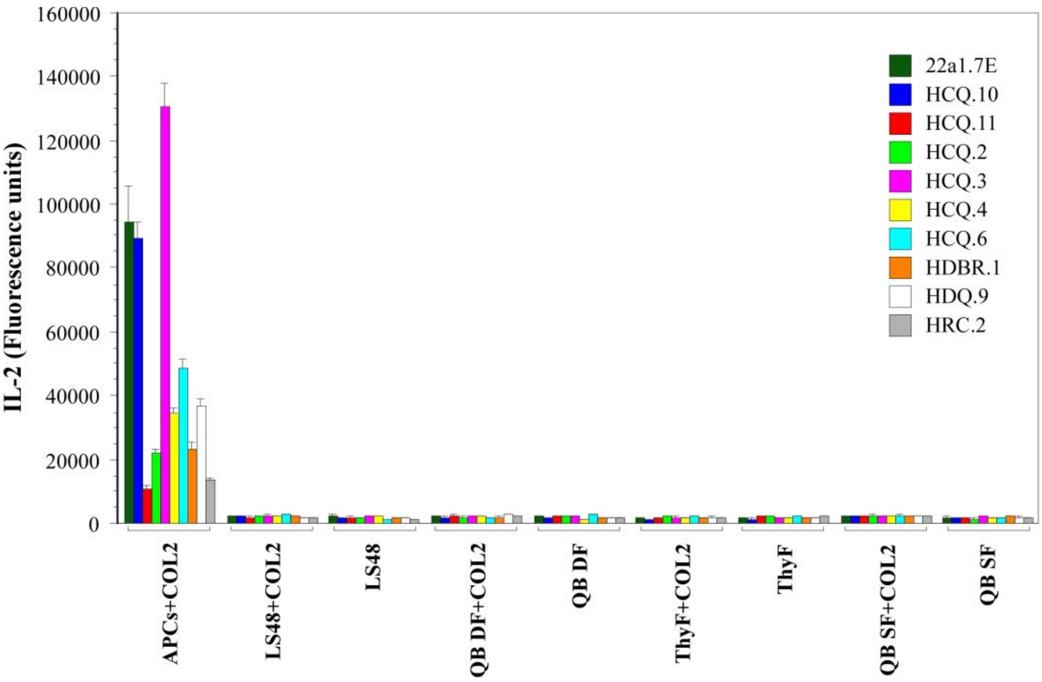

Figure S2. Antigen-presenting capacity of different types of fibroblasts. Antigen-presentation ability of LS48, synovial, dermal and thymic fibroblasts was evaluated by measuring IL-2 levels in culture medium following 24 h co-culture of fibroblasts with COL2-specific T-cell hybridomas and 50 µg/mL COL2. The mean of triplicate measurements for each sample is shown.
